# Spatiotemporal Clustering Analysis of Malaria Infection in Pakistan

**DOI:** 10.3390/ijerph15061202

**Published:** 2018-06-07

**Authors:** Muhammad Farooq Umer, Shumaila Zofeen, Abdul Majeed, Wenbiao Hu, Xin Qi, Guihua Zhuang

**Affiliations:** 1 School of Public Health, Xi’an Jiaotong University Health Science Center, Xi’an 710061, China; rafooq@hotmail.com (M.F.U.); shumailazofeen@yahoo.com (S.Z.); 2 Directorate of Malaria Control Program, Islamabad 44000, Pakistan; amjaffar@hotmail.com; 3 School of Public Health and Social Work, Queensland University of Technology, Kelvin Grove, QLD 4059, Australia; w2.hu@qut.edu.au; 4 Global Health Institute, Xi’an Jiaotong University Health Science Center, Xi’an 710061, China

**Keywords:** malaria, incidence rate, spatiotemporal clustering, spatial cluster analysis, Pakistan

## Abstract

Despite tremendous progress, malaria remains a serious public health problem in Pakistan. Very few studies have been done on spatiotemporal evaluation of malaria infection in Pakistan. The study aimed to detect the spatiotemporal pattern of malaria infection at the district level in Pakistan, and to identify the clusters of high-risk disease areas in the country. Annual data on malaria for two dominant species (*Plasmodium falciparum*, *Plasmodium vivax*) and mixed infections from 2011 to 2016 were obtained from the Directorate of Malaria Control Program, Pakistan. Population data were collected from the Pakistan Bureau of Statistics. A geographical information system was used to display the spatial distribution of malaria at the district level throughout Pakistan. Purely spatiotemporal clustering analysis was performed to identify the high-risk areas of malaria infection in Pakistan. A total of 1,593,409 positive cases were included in this study over a period of 6 years (2011–2016). The maximum number of *P*. *vivax* cases (474,478) were reported in Khyber Pakhtunkhwa (KPK). The highest burden of *P*. *falciparum* (145,445) was in Balochistan, while the highest counts of mixed *Plasmodium* cases were reported in Sindh (22,421) and Balochistan (22,229), respectively. In Balochistan, incidence of all three types of malaria was very high. Cluster analysis showed that primary clusters of *P*. *vivax* malaria were in the same districts in 2014, 2015 and 2016 (total 24 districts, 12 in Federally Administered Tribal Areas (FATA), 9 in KPK, 2 in Punjab and 1 in Balochistan); those of *P*. *falciparum* malaria were unchanged in 2012 and 2013 (total 18 districts, all in Balochistan), and mixed infections remained the same in 2014 and 2015 (total 7 districts, 6 in Balochistan and 1 in FATA). This study indicated that the transmission cycles of malaria infection vary in different spatiotemporal settings in Pakistan. Efforts in controlling *P*. *vivax* malaria in particular need to be enhanced in high-risk areas. Based on these findings, further research is needed to investigate the impact of risk factors on transmission of malaria in Pakistan.

## 1. Introduction

Malaria is a deadly vector-borne infectious disease, rendering around 42% of the world’s population at risk [1]. In the year 2016, malaria resulted in 216 million cases and 445,000 deaths worldwide [2]. Malaria in humans is predominantly due to *Plasmodium falciparum* and *Plasmodium vivax*, which are transmitted mostly by the same species of the genus *Anopheles* [2,3]. Around 99% of malarial deaths were attributed to *P*. *falciparum*, with the majority of them occurring in Africa. *P*. *vivax*, on the other hand, is the dominant cause of malaria in many countries of the Eastern Mediterranean Regional Office (EMRO, Cairo, Egypt) and in the American region [2,4], with 2.5 billion people globally at endemic risk of its morbidity and mortality [5]. Experts believe that in countries having disease load of both *P*. *falciparum* and *P*. *vivax* malaria, the real challenge would be the elimination of *P*. *vivax*, as it has the ability to remain dormant, evading blood-stage treatment and thus causes relapsing infections because of its prolonged neglect based on mistaken assumptions of its harmlessness [6,7]. Mixed *Plasmodium* infection is another significant type of malaria which is usually under-reported, because of over-reporting of the more expressed *P*. *falciparum* or *P*. *vivax* in conventional diagnostic methods [8,9].

Pakistan is classified epidemiologically as a moderate malaria-endemic country with an average national annual parasitic incidence of 1.69%, having diversity within and between its provinces and districts [10]. Pakistan remains one of the highest malaria burden sharing countries in the EMRO region of the World Health Organization (WHO, Geneva, Switzerland), which also includes Sudan, Yemen, Somalia and Afghanistan [2,11]. It is pertinent to note that both *P*. *falciparum* and *P*. *vivax* malaria are prevalent in Pakistan, and incidence of *P*. *vivax* is over 4 times that of *P*. *falciparum* [12]. In 2015, 81% of the total deaths due to *P*. *vivax* outside sub-Saharan Africa took place in only four countries: Ethiopia, India, Indonesia and Pakistan [13]. The coexistence of two species of malaria (*P*. *falciparum* and *P*. *vivax*) in the same community increases the likelihood of mixed *Plasmodium* infections in a single individual [14] and Pakistan is no exception to this, as mixed plasmodium infections have being regularly reported in many parts of the country, making situation more serious [15,16,17].

Malaria has significant impact on developing countries like Pakistan, despite all the disease control strategies designed and executed by the concerned authorities [18,19]. One of the key reasons for this plight is that, epidemiologically, there are not sufficient data and/or robust studies available in Pakistan to evaluate the incidence of malaria [20]. This implies that, to make our efforts more fruitful in controlling malaria, effective surveillance of the disease, as well as information on incidence and/or prevalence of infections or incidence of clinical cases and deaths, are crucial for identifying the most affected population groups to direct resources to these areas for malaria control and prevention [21,22].

Malaria incidence, like many infectious diseases, exhibits spatial and temporal variations with respect to climatic and environmental conditions [1,23]. Thus, the spatial and temporal distribution of malaria—the most basic aspect of its epidemiology—needs to be studied more in the areas of its occurrence [21,24]. Malaria transmission nationwide in Pakistan is seasonal for both *P. vivax* (peaks usually in April) and *P. falciparum* (peaks usually in October) [25]. This characteristic of malaria transmission and distribution may be explored by geographical information system (GIS) mapping and spatial analysis, which are helpful in planning, implementing and measuring impact of disease interventions and then monitoring at subnational, national and regional levels for eventual control of malaria [26,27].

Spatial and spatiotemporal studies on malaria, including its incidence, prevalence, mortality, risk measurement and many other dimensions, have been helpful in many ways in successful design and implementation of programs on malaria control and prevention [24,27]. However, very few studies on the spatial pattern of malaria have been done in Pakistan before. The current study was designed to find out the spatiotemporal pattern of the annual incidence of malaria in Pakistan at the district level, and to detect clusters of high-risk areas of the disease in the country. The results in this study may provide robust evidence for local malaria control and prevention strategy in Pakistan.

## 2. Methods

### 2.1. Study Area

This study aimed to explore malaria cases and incidence at the district level in Pakistan. Geographically, Pakistan is located within the coordinates of 30°00′ N, 70°00′ E, with total land area approximately 796,096 km^2^. According to Pakistan Bureau of Statistics (Census Report 2017), the population of Pakistan is 207.8 million (excluding Azad Kashmir and Gilgit Baltistan) with an average annual growth rate of 2.4% since its last census completed in 1998 [28].

**Figure 1:**
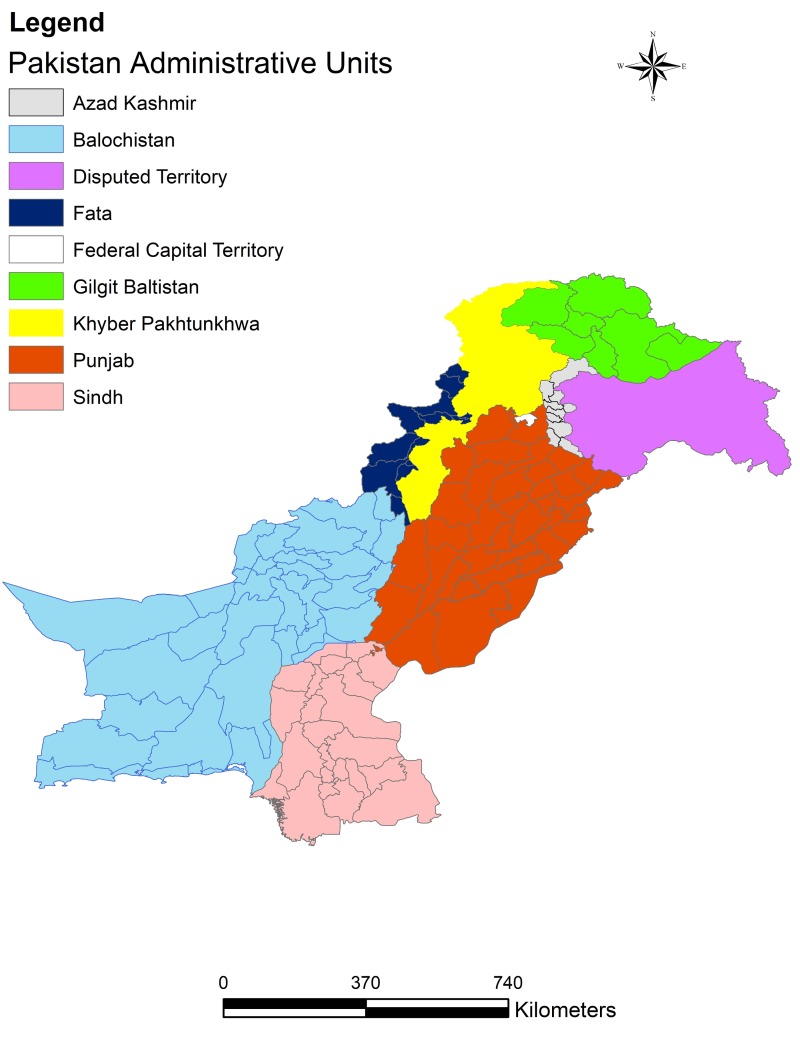
The administrative units in Pakistan.

Pakistan is comprised of the federal capital territory (Islamabad, Pakistan), five provinces (Punjab, Sindh, Khyber Pakhtunkhwa (KPK, Pakistan), Balochistan and Gilgit Baltistan), a group of Federally Administered Tribal Areas (FATA, Peshawar, Pakistan) and an autonomous state (Azad Kashmir, Pakistan). Each of these administrative units is further divided into districts and there is a total of 146 districts in Pakistan. Figure 1 shows the administrative units in Pakistan. Pakistan has diverse climatic, geographic and topographic features, ranging from high snow-covered mountains to hot and dry deserts and moderate plains and coastline [29]. The Human Development Index (HDI) of Pakistan is 0.515, ranking 146 out of 187 countries [10].

### 2.2. Data Collection

The Directorate of Malaria Control Program (DoMC), Islamabad, Pakistan, is the sole federal organization for the national-level planning and policy making to control the disease in the country, and it is implemented by the respective provinces. The aggregated, district-wise annual malaria data by species from 2011 to 2016 were obtained from the DoMC, through a formal request. More details of incidence time (e.g., day and month) and home address (e.g., street number) of malaria cases were not available. All the malaria cases diagnosed at each public sector healthcare facility from individual districts in Pakistan are supposed to report digitally to the Directorate of Malaria Control Program, where data are compiled into a summary sheet. This summary was done at the district level and was used in the current study, which included *P. vivax*, *P. falciparum*, mixed *Plasmodium* and total positive malaria cases, respectively. The national policy with respect to malaria diagnosis and treatment during the study period has been consistent, with improved accessibility of laboratory testing in general. The diagnosis on malaria is made on the basis of WHO-recommended methods of microscopy or rapid diagnostic tests (RDT) [2]. Ten out of 146 districts in Pakistan (mainly Islamabad and districts from Gilgit Baltistan) are not entitled to report cases to DoMC. Thus, the current study represents data from 136 of the total 146 districts in the country. As Gilgit Baltistan has recently been designated as a separate province and the malaria control program was not operational in this part of the country, no data collection or reporting was done there and Gilgit Baltistan was not included in our study. Islamabad, although the capital of the country, is not included in the malaria control program and that is why no data were available on its malaria infection in our study. There are three independent sources which report to DoMC via District Health Information System (DHIS), Malaria Information System (MIS) and Disease Early Warning System (DEWS), but they do not work with the same indicators of malaria. Malaria cases are reported mainly by DHIS, whose reports confirmed cases based on microscopy or RDT. MIS, which was developed recently for the electronic recording of disease statistics, can be used for planning and policy making and contains a few more indicators related to program management and administration than DHIS. DEWS reports the data on suspected malaria cases (not included in our study) and not the confirmed cases diagnosed by microscopy or RDT. The confirmed cases of malaria are mainly reported to DoMC by DHIS, which has its limitations. Firstly, a few of the public sector healthcare facilities do not report to DHIS (security-compromised areas), and secondly, it does not include private healthcare facilities (80% of the patients utilize private healthcare services, which are not regulated and do not have data records [30]), implying that the malaria burden in the country is expected to be 5 to 6-fold more than reported [31,32]. Despite all its limitations, data from DoMC is still the best available, as it is systematically collected by reliable sources and covers almost all parts of the country.

District-wise population data were downloaded from Pakistan Bureau of Statistics, which is based on the latest census completed in 2017. This district-wise population of 2017 was taken as a reference and it was retrospectively extrapolated, depreciating by 2.4% annually, which is the average annual growth rate of the country between 1998 and 2017.

### 2.3. Data Analyses

Statistical analyses were performed by steps. Firstly, malarial cases data and population data were compiled in Microsoft Excel according to the requirements of the GIS software. Then, the annual incidence rate (IR) and relative risk of each species of malaria cases (*P*. *vivax*, *P*. *falciparum*, mixed *Plasmodium* and total positive) at the district level were calculated using the mean malaria incidence rate of the whole country by species in each year as the reference. Using the relative risk at the district level can help quantify district IR compared to the national level. The descriptive statistics of IR and sequence plots of annual cases for each species of malaria were performed using statistical package SPSS 13.0 [33].

The aligned annual malaria cases data by species, along with annual population and latitude/longitude coordinates of centroids of districts, respectively, were input in SaTScan 9.4.4 for the spatial cluster analysis. SaTScan is used to perform spatial, temporal and space–time analysis using scan statistics. This software has widespread application in fields like epidemiology, ecology, economics, geography and many others [34]. In the field of health and epidemiology, SaTScan has been used to detect spatial and space–time disease clusters and to evaluate the statistical significance of these clusters for geographical surveillance of the disease [35,36]. In our study, purely spatial scanning analysis was performed for clusters with high rates using the discrete Poisson model. The clusters included primary clusters (the cluster with highest risk among all clusters in the map) and secondary clusters (other statistically significant high-risk clusters) in each year from 2011 to 2016, identified as a result of SaTScan analysis. The selection of “No geographic overlap” was used as a default setting so that secondary clusters may not overlap the most significant (primary) cluster. The maximum spatial cluster size on the spatial window was set to be at 10% of the population at risk, and the maximum radii of the reported clusters was set to be 200 km from the SaTScan setting, based on the total land area and population distribution in Pakistan. A similar method of setting maximum population size and cluster radii has also been used in another spatial cluster study [36]. Relative risk of high-risk clusters of each species of malaria for each year was estimated using the Poisson model and by SaTScan, considering case number and population size of districts within each cluster. The relative risk was then plotted in an Excel sheet containing the aligned data.

The spatial patterns of malaria IR, the relative risk and the information on primary and secondary clusters by species at the district level (2011–2016) were displayed in maps using ArcGIS 10.2 [37]. This study obtained ethical approval from all the relevant authorities. The approval letter for malaria by species data was obtained from Directorate of Malaria Control Program Pakistan, then an Exemption approval letter was obtained from National Bioethical Committee Pakistan, letter number No.4-87/NBC-279-Exempt./17/1139. The research protocol was approved by Institutional Review Board of Xi’an Jiaotong University, China.

## 3. Results

A large number of malaria cases by species were reported across the country during the period of this study (2011–2016). We found 1,593,409 total positive cases in the 6-year study period in Pakistan, with highest burden in KPK. A total of 1,221,026 cases of *P*. *vivax* were reported, with the majority of cases being found in KPK (474,478). There were 306,172 *P*. *falciparum* cases, with the highest number of cases in Balochistan (145,445). The mixed *Plasmodium* cases totaled 66,211, with Sindh (22,421) and Balochistan (22,229) being the top contributors (both provinces reported almost equal numbers of mixed *Plasmodium* cases).

Table 1 explains the IR (per 100,000) of malaria by species at the district level during the study period. The incidence of malaria by species varies considerably among the districts and over the years. Standard deviation is higher than the respective mean of IR for each species of malaria in each year of the study period, showing disparities in disease incidence among the districts in Pakistan, and this difference is further evidence of the wide gaps amid all the minimum and maximum values of IR in this table. IR of *P*. *vivax* malaria was higher than the other two species (*P*. *falciparum* and mixed *Plasmodium* malaria) at the district level in each year of the study period, except in 2011, when the IR of *P*. *falciparum* remained the highest of all three malaria species at the district level.

**Figure 2:**
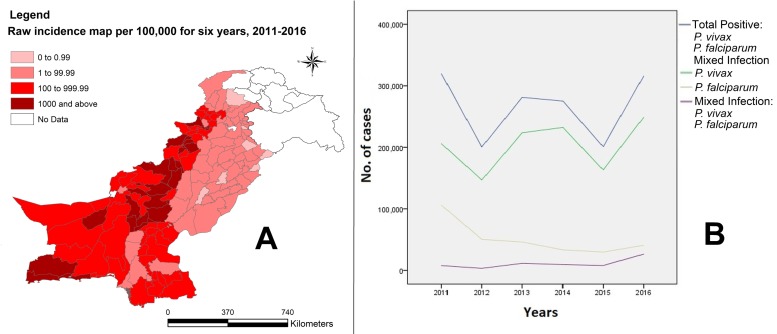
(**A**) Raw incidence (per 100,000) of aggregated total positive cases at the district level in Pakistan, and (**B**) sequence plot of annual cases of malaria by type in Pakistan, 2011–2016.

Figure 2 shows the IR of aggregated total positive cases from 2011 to 2016 and represents a summary of the disease burden in the country at the district level during the whole study period (Figure 2A). We divided IR into five categories to demarcate the districts with no data and the ones with high to very high and low to very low IR of the disease. It also shows the sequence plot of annual cases of malaria by species, 2011–2016. This figure upholds the findings in Table 1, as it confirms the annual variations in burden of malaria in the country. Figure 2B exhibits that the case counts for total positive and *P*. *vivax* are in close proximity to one another and look alike, implying that *P*. *vivax* is the principal burden bearer of malaria in the country and is responsible for annual variation in the disease load. The figure also demonstrates that *P*. *falciparum* infection is declining with the passage of time, while mixed *Plasmodium* has a potential rising trend.

**Figure 3:**
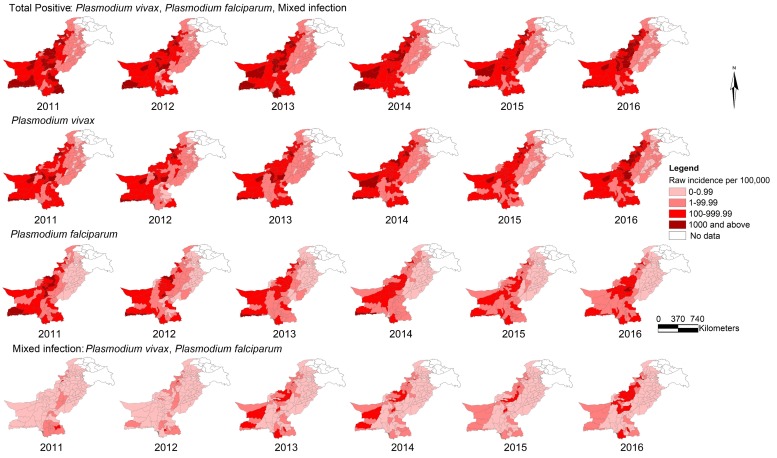
Raw incidence (per 100,000) of malaria by type at the district level in Pakistan, 2011–2016.

Figure 3 illustrates the IR (per 100,000) of malaria by species at the district level, 2011–2016. In both Figure 2 and Figure 3, the darker shades in the geographical maps indicate districts with higher IRs of malaria, while the lighter shades represent the ones with lower IRs. Figure 3 expounds on Figure 2 in detail, thus conveying that IR of *P*. *vivax* malaria in Pakistan is much higher than that of *P*. *falciparum* and mixed *Plasmodium* malaria, and the patterns of distribution of total positive and *P*. *vivax* malaria in the corresponding years were similar. This figure further illustrates that total positive and *P*. *vivax* malaria with high IRs are concentrated in the south, west and northwest parts of Pakistan and that *P*. *falciparum* malaria is shrinking over the years, while mixed *Plasmodium* is gradually expanding.

**Figure 4:**
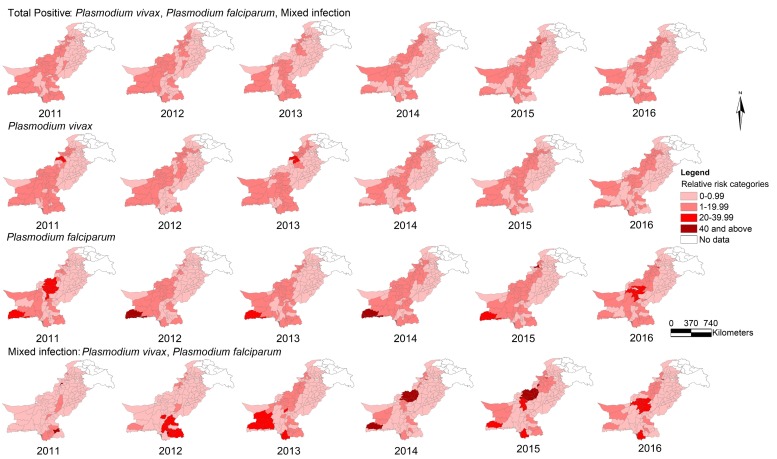
Relative risk of malaria by type at the district level in Pakistan, 2011–2016.

Figure 4 displays the relative risk of malaria by species at the district level from 2011 to 2016. The distribution patterns of relative risk (RR) of total positive and *P*. *vivax* malaria in the corresponding years look alike and are largely in medium categories (1–19.99), covering most districts of the country. RR values of *P*. *falciparum* malaria are mostly in medium to high categories (1–19.99 and 20–39.99) and RR values of mixed *Plasmodium* malaria attained peak values (RR 40 and above) in a few districts.

**Figure 5:**
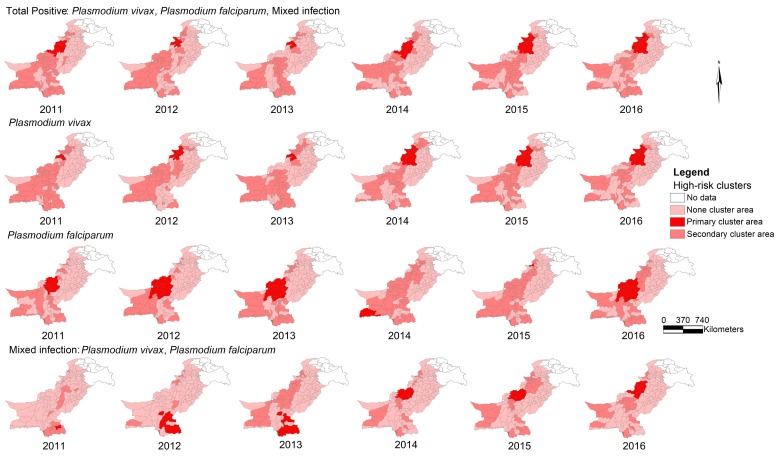
High-risk clusters of malaria by type at the district level in Pakistan, 2011–2016.

Figure 5 exhibits the high-risk clusters of malaria by species at the district level from 2011 to 2016. This figure displays most significant (primary) and secondary clusters of the disease and the “no cluster areas” in the geographical maps. Once again, there is a similarity in the patterns of distribution of total positive and *P*. *vivax* disease clusters.

Table 2 shows details about most significant high-risk districts (primary clusters) of the disease during the study period. It gives yearly information on number of districts, coordinates of centroid, radii, population, number of cases, IR, relative risk and statistical significance of the primary clusters of malaria by species. It was established that the number of cases and IRs of *P*. *vivax* malaria were much higher than those of the other two species of malaria (*P*. *falciparum* and mixed *Plasmodium*) in the respective years. Another important finding is that the districts (number and locations) in primary clusters of (i) *P*. *vivax* are same for 2014, 2015 and 2016 (total 24 districts, 12 in FATA, 9 in KPK, 2 in Punjab and 1 in Balochistan); (ii) *P*. *falciparum* are same for 2012 and 2013 (total 18 districts, all in Balochistan); (iii) mixed *Plasmodium* are same for 2014 and 2015 (total 7 districts, 6 in Balochistan and 1 in FATA). We also tested spatial cluster analysis using other parameters (100 km, 300 km and 400 km for maximum radius of cluster, and 5%, 15% for maximum population at risk) and did not find significant differences from the above results. 

## 4. Discussion

In this study, the incidence of malaria by species in Pakistan fluctuated among districts, which is in accordance with previous findings [38]. Our study confirmed the occurrence of three species of malaria (*P*. *vivax*, *P*. *falciparum* and mixed *Plasmodium*) in the country, and incidence of *P*. *vivax* malaria was significantly higher than for the other two species [39,40]. The increase in the incidence of *P*. *vivax* in recent years may be due to the focused efforts of the malaria control program on case detection. Most of the malaria high endemic districts were found in Balochistan, FATA and KPK, with Sindh estimated as a moderately high endemic area. These findings are similar to those in past studies conducted in Pakistan [11,41]. According to our study, spatial distribution of the most significant high-risk clusters of malaria by species exhibited that *P*. *vivax* primary clusters comprised districts in FATA and KPK, *P*. *falciparum* primary clusters were mainly in Balochistan, while mixed *Plasmodium* primary clusters included districts in Sindh and Balochistan. In general, the pattern of malaria incidence remained consistent over the study period. The finding in our study that the same districts persisted in primary clusters in different years can be useful for prompting policy makers to involve authorities from different administrative units to put combined efforts in combating the disease.

The high incidence of malaria by species in FATA, Balochistan and, to some extent, in KPK, is explained by the fact that most of the districts in these areas are lagging behind in their social and living standards compared to the rest of country [42], which is due to compromised the law and order situation in these areas, resulting in very low-performing health facilities (e.g., limited health care resources and unavailability to health facilities for diagnosis and treatment in some communities), therefore increasing malaria in particular, and other diseases in general, in these areas [31,43]. In Balochistan, alarmingly, incidence of all three species of malaria was very high, demanding contemplation by the concerned authorities. In some districts of Balochistan, *P*. *vivax* malaria was dominant, as supported by the evidence from other studies [44,45], whereas, in its eastern and southeastern districts, *P*. *falciparum* malaria burden was higher [46,47]. In the northern parts adjoining KPK, both *P*. *vivax* and *P*. *falciparum* malaria coexisted in large numbers [48]. Moreover, in this study, Balochistan, along with Sindh, reported the highest burden of mixed *Plasmodium* malaria, which reiterates the presence in abundance of both *P*. *vivax* and *P*. *falciparum* malaria in these areas. In most districts of KPK, *P*. *vivax* turned out to be higher than *P*. *falciparum* and mixed *Plasmodium* malaria, as was shown in the other studies [15,49,50]. Sindh was estimated as a moderately high malaria-endemic area in this study, with some high-risk districts adjoining Balochistan and a few near coastal areas [51].

The hypothesis that *P*. *falciparum* and *P*. *vivax* are endemic in separate regions can be attributed to the variability of climatic conditions, proximity to or remoteness from the coastal line and particularly due to antimalarial drug resistance [40,52]. Incidence of *P*. *falciparum* malaria is higher in Balochistan and Sindh, where chloroquine drug resistance may have contributed to this species’ dominance and, also, these two provinces have a coastal line in proximity to many of its districts [40,52]. The use of the latest antimalarial drugs, like artemisinin-based combination therapy, could help address the problem [40,52]. On the other hand, *P*. *vivax* is endemic throughout the country, including Sindh and Balochistan, and chloroquine is still considered the first-line drug therapy against *P*. *vivax* [40,52].

*P*. *vivax* and mixed *Plasmodium* malaria incidences were reasonably high in many districts of Sindh, which is a serious concern, whereas *P*. *falciparum* malaria showed a decline over the years in this province in the course of this study. Punjab was assessed as a low malaria-endemic area in this study, despite the fact that it covers 53% of the total population in Pakistan. The lower incidence of malaria by species in Punjab could be attributed to the fact that, firstly, during the last three decades, ecological changes (waterlogging and salinization) resulted in selection of the fittest yet less efficient vector species in this province [53] and, secondly, because of better healthcare delivery infrastructure than the rest of country due to better social and living standards here [52]. It is important to mention that, although overall incidence rate of malaria by species was significantly low in Punjab, southern districts had higher IRs than the central and northern ones within the province, thus requiring attention by the authorities [19].

In Pakistan, an estimated 20% of the population consult public sector healthcare facilities for their health needs. These health-seeking statistics are not uniform throughout the country but have been found varying in different provinces. According to Pakistan Social and Living Standards Measurement Survey 2014–2015, 17% of the people visited public sector health facilities for consultation and/or treatment in Punjab, 20% in Sindh, and 28% each in Khyber Pakhtunkhwa and Balochistan. These percentages suggest that the better the living standards in a province, the more their paying capacity and, thus, the more its people seek healthcare from private sector health facilities. The living standards and therefore paying capacity of Balochistan and KPK are relatively lesser as compared to other provinces, hence malaria burden in these areas is higher. Moreover, this also reflects the performance of public sector health facilities in KPK and Balochistan that despite these facilities are being used by the community the malaria burden is increasing. As no adjustments were made in this study for the malaria burden reported by public sector health facilities versus the private sector ones, so considering the higher percentages of population utilizing private sector healthcare facilities, it is important to mention here that estimates shown here are to be shown patterns of incidence rather than absolute values. 

This study has several strengths to its credit. This is the first study in Pakistan exploring spatiotemporal pattern and high-risk clusters of malaria incidence at the district level. Latest data (malaria by species and population data) were used in this study to describe current patterns of disease. The study encompassed almost whole country at the district level and discussed all three species of malaria infections incidence in Pakistan. This study provides novel information on the malaria burden in Pakistan with respect to time and space discovering malaria hotspots at the district level. The results in this study may provide scientific evidence for malaria control and prevention strategies in Pakistan. 

Our study had certain limitations as well that can be taken into account in designing the subsequent studies. This study caters only those patients who consulted public sector healthcare facilities (20% of the total population) and gives no information about the larger section of population going to private sector healthcare facilities. Thus, the actual malarial incidence may be underestimated. The data did not describe individual patients’ profiles (age, sex, socioeconomic and education status, etc.), as aggregated data was used in this study. The data we collected from DoMC was aggregated annual malaria data, and month and day of malaria incidence at the district level were not available. Thus, seasonality of malaria at the district level in Pakistan could not be established. Geographical boundaries of districts were consistent in the study period, but the aggregated malaria data at the district level have no more specific geographical details, e.g., home address information. Thus, the results at the district level (including using centroid of districts to detect spatial clusters by SaTScan) may have bias from a geographical perspective, e.g., masking the potential impact of socioeconomic disparity across different communities on malaria incidence within each district. Moreover, the exact neighborhood of each district, which is significant in influencing distribution of clustering districts, cannot be reflected in SaTScan analysis.

Some recommendations can be proposed based on the findings of this study. *P*. *vivax* malaria may no longer be considered as benign, keeping in view the morbidity and mortality associated with it [5]. Also, the number of hospitalizations with *P*. *vivax* infection have increased over time, and mortality rates attributable to both species of malaria (*P*. *vivax* and *P*. *falciparum*) in our part of the world are comparable [54,55]. As the diagnosis of mixed *Plasmodium* malaria is becoming more challenging now, owing to the conventional and sometimes poor microscopy practices, enhancing microscopy skills through trainings and, if feasible, bringing new diagnostic techniques, like polymerase chain reaction (PCR), into practice may improve the diagnosis [17,39]; although costly, PCR helps in precise detection of mixed infections. Irrational and high use of antimalarial drugs, as reported in different studies [40,52], has contributed to drug-resistant malaria in Pakistan, which should be noticed by authorities before the threat gets too serious to tackle. The knowledge and practices about malaria and its prevention are seriously deficient among the people of Pakistan, especially those living in rural settings, and this aspect requires work on a large scale for prospects of success in controlling malaria in Pakistan [56,57]. The high burden of malaria needs the attention of authorities to locate the high-risk areas and then direct resources and interventions (long-lasting insecticidal nets, sprays, drugs against malaria, community awareness campaigns) towards these areas by involving local community and local authorities for long-lasting control and, eventually, elimination of the disease. The control strategies are already in place in Pakistan, including in the areas of its high incidence, but due to limited supplies and sometimes inequitable distribution, disease control and prevention still needs to be improved. Public sector healthcare facilities need to deliver better services to the community, as they are utilized mostly by the underprivileged ones. The private sector healthcare facilities should be regularized by legislative bodies to streamline their performances by maintaining and sharing data with the malaria control program, thus controlling the disease in totality, including the disease-reporting system. More public health investigations and studies need to be conducted in hotspot areas explored in our study in Pakistan. Besides, seasonality of malaria and its environmental drivers (e.g., climate change and extreme weather) are important aspects in the pattern of disease development, yet very few detailed studies have been reported in Pakistan [58]. Thus, this should be explored for better understanding of the disease progression in the country.

## 5. Conclusions

This study described spatial and temporal variations in all three species of malaria widespread in Pakistan at the district level. As a result, high-risk districts of malaria by species were identified by conducting spatial cluster analysis, which has its significance in exploring area-specific evidence on disease progression. The study results suggested ramping up malaria control activities in Balochistan, FATA, KPK and Sindh, which are high burden disease areas. Diagnosis of mixed *Plasmodium* malaria is a challenge to be taken seriously, keeping in mind its high-risk areas (i.e., Balochistan and Sindh). Efforts in controlling *P*. *vivax* malaria need to be enhanced, since it is the major burden bearer in most parts of the country, while, simultaneously, not being complacent about *P*. *falciparum* malaria, which is still causing damages in the country, especially in Balochistan. More studies are required with detailed spatial and temporal analysis in the individual high-risk districts for further exploration of patterns in malaria progression and possible risk factors, for its control and prevention in Pakistan and its regions.

## Figures and Tables

**Table 1:** Summary of descriptive statistics of malaria incidence rate (per 100,000) by type at the district level in Pakistan, 2011–2016.

Year	*Plasmodium vivax*		*Plasmodium falciparum*		Mixed *Plasmodium*		Total Positive	
	Mean ± SD	Min–Max	Mean ± SD	Min–Max	Mean ± SD	Min–Max	Mean ± SD	Min–Max
2011	264.43 ± 523.15	0–3745.63	181.17 ± 560.99	0–5322.22	7.26 ± 47.13	0–523.01	452.85 ± 866.79	0–5903.79
2012	259.12 ± 629.89	0–4913.11	109.42 ± 265.42	0–1811.71	4.25 ± 19.66	0–189.07	372.79 ± 786.09	0–5805.01
2013	439.90 ± 1528.29	0–15,615.63	99.04 ± 251.98	0–2289.91	28.75 ± 105.69	0–1112.85	568.09 ± 1826.10	0–19,018.40
2014	430.25 ± 1202.55	0–11,509.69	70.54 ± 169.64	0–1198.92	26.18 ± 80.30	0–525.08	526.98 ± 1338.77	0–12,442.91
2015	278.85 ± 584.71	0–3813.32	63.42 ± 155.33	0–1085.41	21.47 ± 74.44	0–505.52	363.76 ± 733.58	0–4335.27
2016	617.27 ± 2096.72	0–19,935.05	98.61 ± 245.61	0–1914.28	70.93 ± 215.63	0–1687.26	786.18 ± 2434.84	0–22,071.55

Mixed *Plasmodium*: *Plasmodium vivax*, *Plasmodium falciparum*. Total positive: *P. vivax*, *P. falciparum* and mixed *Plasmodium*.

**Table 2:** Summary of primary high-risk clusters’ information of malaria by type at the district level in Pakistan, 2011–2016.

Type of Malaria	Year	Number of Districts in Primary Cluster	Coordinates of Primary Cluster Centroid	Primary Cluster Radius (km)	Primary Cluster Population	Number of Cases in Primary Cluster	Incidence Rate/100,000	RR	*p* Value
*Plasmodium vivax*	2011	5	33°00′ N, 70°44′ E	59.53	2,175,181	43,211	1987.9	22.61	<0.0001
	2012	17	33°69′ N, 70°31′ E	142.42	11,009,128	63,356	574.3	11.29	<0.0001
	2013	8	32°65’N, 70°39′ E	49.46	2,725,805	59,438	2182.0	23.45	<0.0001
	2014	25	33°00′ N, 70°44′ E	181.76	18,172,987	113,900	627.2	8.76	<0.0001
	2015	25	33°00′ N, 70°44′ E	181.76	18,705,115	76,566	409.6	7.98	<0.0001
	2016	25	33°00′ N, 70°44′ E	181.76	19,253,991	136,436	707.1	10.96	<0.0001
*Plasmodium falciparum*	2011	8	30°30′ N, 68°72′ E	118.93	1,293,463	21,447	1659.2	33.96	<0.0001
	2012	19	30°04′ N, 67°82′ E	195.57	5,220,346	18,683	357.1	19.17	<0.0001
	2013	19	30°04′ N, 67°82′ E	195.57	5,412,261	11,211	207.3	10.33	<0.0001
	2014	2	25°40′ N, 63°43′ E	75.41	646,999	4947	765.1	49.20	<0.0001
	2015	1	34°04′ N, 71°20′ E	0	854,777	4978	582.8	44.10	<0.0001
	2016	19	30°04′ N, 67°82′ E	195.57	6,039,018	13,472	222.6	15.31	<0.0001
Mixed *Plasmodium*	2011	8	29°98′ N, 69°54′ E	153.44	7,719,217	13,752	178.3	22.21	<0.0001
	2012	13	25°98′ N, 69°31′ E	174.87	14,988,376	2474	16.5	33.96	<0.0001
	2013	12	25°34′ N, 69°75′ E	190.84	12,975,991	4289	33.1	7.63	<0.0001
	2014	7	31°22′ N, 68°78′ E	137.22	1,173,936	3008	256.4	72.87	<0.0001
	2015	7	31°22′ N, 68°78′ E	137.22	1,205,587	1658	137.6	40.95	<0.0001
	2016	14	32°29′ N, 69°81′ E	154.61	6,303,983	9941	157.4	17.96	<0.0001
Total positive	2011	13	32°29′ N, 69°81′ E	153.66	5,289,934	76,418	1445.6	9.96	<0.0001
	2012	9	33°00′ N, 70°44′ E	77.62	3,986,266	49,846	1247.9	14.19	<0.0001
	2013	7	32°65′ N, 70°39′ E	49.46	2,725,805	66,089	2426.2	19.85	<0.0001
	2014	15	32°29‘ N, 69°81′ E	158.42	6,447,603	81,991	1272.5	11.67	<0.0001
	2015	25	33°00′ N, 70°44′ E	181.76	18,705,115	88,869	475.4	7.17	<0.0001
	2016	25	33°00′ N, 70°44′ E	181.76	19,253,991	157,613	816.9	9.00	<0.0001

RR: relative risk. Mixed *Plasmodium*: *P. vivax*, *P. falciparum*. Total positive: *P. vivax*, *P. falciparum* and mixed *Plasmodium*.

## Abbreviations

P	*Plasmodium*
EMRO	Eastern Mediterranean Regional Office
WHO	World Health Organization
GIS	Geographical Information System
KPK	Khyber Pakhtunkhwa
FATA	Federally Administered Tribal Areas
DoMC	Directorate of Malaria Control Program
DHIS	District Health Information System
MIS	Malaria Information System
DEWS	Disease Early Warning System
IR	Incidence Rate
RR	Relative Risk
PCR	Polymerase Chain Reaction
